# Phenotypical profile and global transcriptomic profile of Hypervirulent *Klebsiella pneumoniae* due to carbapenemase-encoding plasmid acquisition

**DOI:** 10.1186/s12864-019-5705-2

**Published:** 2019-06-11

**Authors:** Dan Long, Lan-lan Zhu, Fang-ling Du, Tian-xin Xiang, La-Gen Wan, Dan-dan Wei, Wei Zhang, Yang Liu

**Affiliations:** 1 Department of Clinical Microbiology, First Affiliated Hospital of Nanchang University, Nanchang University, Yong wai zheng jie No. 17, Nanchang, 330006 People’s Republic of China; 2 Department of Respiratory, First Affiliated Hospital of Nanchang University, Nanchang University, Yong wai zheng jie No. 17, Nanchang, 330006 People’s Republic of China

**Keywords:** Hypervirulent *K. pneumoniae*, Transcriptomic analysis, Carbapenemase plasmid, RNA-Seq technique

## Abstract

**Background:**

Plasmids play an vital role in driving the rapid global spread of antimicrobial resistance and adaptation to changing ambient conditions. It has been suggested that the presence of plasmids can pose tremendous impacts on the host physiology. However, little is known regarding the contributions of carbapenemase-encoding plasmid carriage on the physiology and pathogenicity of hypervirulent *K. pneumoniae* (hvKP).

**Results:**

Here we performed a transcriptomic analysis of hvKP with or without carbapenemase-encoding plasmid p24835-NDM5. The results had shown 683 genes with differential expression (false discovery rate, ≤0.001; > 2-fold change), of which 107 were up-regulated and 576 were down-regulated. Gene groups with functions relating to carbohydrate metabolism and multidrug efflux system were increased in genes with increased expression, and those relating to capsule biosynthesis and virulence factors were increased in the genes with decreased expression. In agreement with these changes, survival rate of TfpNDM-hvKP in the presence of normal human serum decreased, and competitive index (CI values) indicated significant fitness defects in the plasmid-carrying hvKP strain when co-cultured with its plasmid-free isogenic ancestor and the ATCC control. Moreover, the p24835-NDM5-containing hvKP strain retained its high neutrophil-mediated phagocytosis and murine lethality.

**Conclusion:**

These data indicate that hvKP responds to carbapenemase-encoding plasmid by altering the expression of genes involved in carbohydrate metabolism, antibiotic resistance, capsule biosynthesis and virulence expression. Apart from antibiotic resistance selective advantages, carbapenemase-encoding plasmid carriage may also lead to virulence change or adaption to specific habitats in hvKP strain.

**Electronic supplementary material:**

The online version of this article (10.1186/s12864-019-5705-2) contains supplementary material, which is available to authorized users.

## Background

A newly emerged superbug, hypervirulent *Klebsiella pneumoniae*, occurs frequently worldwide, which may post enormous threat to human health. Recently, carbapenem-resistant and hypervirulent *Klebsiella pneumoniae* (CR-hvKp) strains have emerged while therapeutic options remain limited. [1] Producing carbapenemases is an important mechanism of *Klebsiella pneumoniae* in carbapenem resistance. The worldwide dissemination of many carbapenemase genes is largely due to conjugative plasmids. One such carbapenemase, the New Delhi metallo-β-lactamase carbapenemase (NDM), was first detected in a *Klebsiella pneumoniae* isolate from a Swedish patient previously hospitalized in India [2]. It is now acknowledged that the NDM-producing *Klebsiella pneumoniae* has become endemic in several regions worldwide. The most predominant *bla*_NDM_-carrying plasmids among *K.pneumoniae* isolates belonged to the IncX3 group, which includes p24835-NDM5 [3]. The plasmid most often associated with carbapenem resistance in CR-hvKp isolates in China was a conjugative 46,161-bp IncX3-like plasmid. The *bla*_NDM_ gene is located within an ISAba125 and IS26 flanked transposon-like element. Sequencing of p24835-NDM5 (GenBank accession no. CP014006) revealed high similarity to pNDM-MGR194 (GenBank accession no. KF220657) and pNDM-HN380(GenBank accession no. JX104760), which were found in India and China, respectively [3].

Horizontal gene transfer of carbapenemase plasmids is an important factor in evolution of CR-hvKP [1]. However, successful dissemination of resistance plasmids largely depends on the fitness cost imposed on hosts [4]. Previous studies have proved the strong impacts of plasmid on the host physiology and cellular metabolism, including alterations in transcription and translation in *Escherichia coli* [5–7]. Therefore, it is crucial to characterize the phenotypical and transcriptional profile of hvKP strain before and after acquiring plasmids. In this study, to elucidate the nature of alterations usually known as ‘perturbation’ of hvKP cell function by carbapenemase-encoding plasmid carriage, we performed comprehensive comparision of the virulence and transcriptomic expression between the p24835-NDM5-containing hvKP strain and the p24835-NDM5-free strain. This study provides the first insight into hvKP gene expression regulated actively by carbapenemase-encoding plasmid carriage.

## Methods

### Bacterial strain, plasmid, and strain construction

*K. pneumoniae* BD2411 (ST23; capsule serotype K1) served as a recipient in transformation experiment. The hypervirulent strain BD2411 was recovered from a blood sample from a 28-year-old Chinese male in the teaching hospital of Nanchang University, China, in 2015, who developed pyogenic liver abscess with metastatic spread to the spleen. The isolate was subjected to a string test to identify it as a hypermucoviscosity (HMV) *K. pneumoniae* as previously described. [8] The genomic DNA of strain BD2411 was sequenced using an Illumina HiSeq 2000 instrument, generating 4,953,798 bp, with a GC content of approximately 58.40%. The results from PlasmidFinder showed that it had a pLVPK-like virulence plasmid, including the region in which the *rmpA2*, *iucABCD*, *iroBCDN* and *iutA* genes were located. A clinical *K. pneumoniae* isolate containing a ca. 46-kb sized IncX3-type plasmid (p24835-NDM5) served as a donor of *bla*_NDM-5_ (Additional file 1) [3]. The annotated plasmid sequence of p24835-NDM5 was deposited in the GenBank database under accession number CP014006. Transformation of BD2411 with plasmid p24835-NDM5 resulted in strain TfpNDM-hvKP. Positive transformant was confirmed by PCR and DNA sequencing with specific primer sequences to serotype K1 and *bla*_NDM_ gene.

### Antimicrobial susceptibility testing

The susceptibility of the transformant TfpNDM-hvKP and its plasmid-free isogenic ancestor to antimicrobial agents was performed initially using Gram-negative susceptibility cards on the Vitek system (bioMerieux, France). Minimum inhibitory concentrations (MICs) for the clinically important antimicrobial agents (ampicillin, amikacin, gentamicin, levofloxacin, piperacillin/tazobactam, cefotaxime, ceftazidime, cefepime, aztreonam, cefoxitin, imipenem, meropenem, ertapenem and trimethoprim/sulfamethoxazole) were further determined by using the the microdilution method in accordance with the Clinical and Laboratory Standards Institute (CLSI) guidelines [9]. *Escherichia coli* ATCC 25922 and *K.pneumoniae* ATCC700603 were used as a quality control reference strain for antimicrobial susceptibility testing.

### Plasmid transformation and stability

Plasmid p24835-NDM5 was purified by a commercial kit (Qiagen, USA) and used to transform electrocompetent recipient *K. pneumoniae* BD2411. Briefly, 10 μl of plasmid DNA (50 ng) was mixed into 100 μl of electrocompetent *K.pneumoniae* BD2411 recipient in a microcentrifuge tube. After placing the mixture on ice for 30 min, the tube was placed into a 42 °C water bath for 45 s and then put back on ice for 2 min. Luria-Bertani broth was added into the tube and the mixture grew in 37 °C shaking incubator for 45 min. Transformants were selected on LB agar with 0.5 μg/ml of imipenem. Southern blotting was performed on agarose gel by in-gel hybridization with the *bla*_NDM_ probe labeled with digoxigenin DIG-high prime labeling mix (Roche, Germany) detection Kit. Plasmid DNA from transformant was separated by S1-PFGE and was transferred to nylon membrane (Hybond N, Amersham, UK), hybridized with prepared probes.

Stability of p24835-NDM5 was conducted as described previously [10]. Plasmid stability was estimated by propagating monocultures for ∼100 generations as well as of the transformant in 1:1000 dilutions without any antibiotic pressure. One hundred colonies were selected on LB agar with 0.5 μg/ml imipenem. Plasmid presence was checked by colony PCR targeting *bla*_NDM-5_ and *repB* or *repA*. The stability test was performed three times, and the average values are presented.

### Growth kinetics

Non-competitive growth experiments on WT and TfpNDM-hvKP strains were performed in 96-well microtiter plates. Strains were grown at 37 °C in brain-heart infusion (BHI) broth (Oxoid, UK) until an optical density at 600 nm (OD600) of 0.2 was reached. For each growth curve measurement, the strains were grown with shaking (180 rpm). Bacterial growth was monitored by measuring the OD600 until until the cultures reached stationary phase (EXL808, Biotek America). Detection interval time is 30 minuntes.

### Competition experiments and fitness estimation

To determine the effect of p24835-NDM5 carriage on bacterial fitness, pairwise competition experiments in vitro were carried out using the TfpNDM-hvKP strain competed with its plasmid-free isogenic ancestor BD2411. Competition experiments were set up and carried out as previously described. [11] Initial and final titres of each competitor were determined by selective (100 mg/L ampicillin) and nonselective plating and the relative fitness calculated. The competitive indexes (CIs) value are shown as the ratios of the CFUs of the p24835-NDM5-containing strain to CFUs of its plasmid-free isogenic ancestor. By definition, a ratio greater than or less than 1 indicates increased or decreased fitness, respectively. The relative fitness cost of clinically isolated *K. pneumoniae* BD2411 was compared with a control strain *K. quasipneumoniae* subsp*. similipneumoniae* strain ATCC 700603. Fitness cost of plasmid carriage compared with the plasmid-free ancestor and control strain was assessed with one-sample t-tests. Statistical analysis was performed in R (https://www.r-project.org/).

### Extraction and quantification of capsule

To evaluate capsule production by *K. pneumoniae* strains, uronic acid content was extracted and quantified, as previously described. [12] Briefly, cultures (500 μL) were mixed with 100 μL 1% zwittergent-100 mM citric acid (Sigma-Aldrich, USA), and then incubated for 20 min at 50 °C. After centrifugation (13,000 rpm for 15 min), 300 μL of the resulting supernatants were precipitated with 1.2 ml 100% ethanol. Following centrifugation, pellets were dried and resuspended in 200 μL of distilled water, and then 1.2 ml sodium tetraborate-concentrated H_2_SO_4_ was added to each sample. The reaction mixture was subjected to vortex mixing and boiled for 5 min. 3-hydroxydiphenol (Sigma-Aldrich, USA) was added to a final proportion of 0.15% (vol/vol). After a 5-min incubation at room temperature, the absorbance at 520 nm was then measured. The amounts of capsule production was determined from a standard curve of glucuronate lactone (Sigma-Aldrich, USA).

### Virulence studies

Virulence assessments were performed by serum resistance, neutrophil phagocytosis and mice lethality assay. Neutrophil phagocytosis and serum resistance were performed as previously described. [3, 13] To determine the LD_50_ in a murine model, 6 to 8 week old BALB/c mice with an average weight of 20–25 g were purchased from Nanchang University Animal Center. Ten-fold serial dilutions of bacteria (three strains: WT, its transformant TfpNDM-hvKP, and the ATCC control) were prepared from a starting concentration of 10^6^ colony forming unit (CFU)/ml to final concentration 10^2^ CFU/ml with 5 concentrations, and each dilution was injected intraperitoneally into the adult BALB/c mice [14]. Inoculated mice were observed for signs and symptoms of infection for 14 days. Survival rate of the inoculated mice was recorded daily. The LD50 was calculated as described by Reed and Muench [15]. Survival curves were obtained by the Kaplan-Meier method and compared by the log-rank test. A *P* value of less than 0.05 was considered to indicate statistical significance. All animal work was performed in conformity with NIH guidelines (NIH Pub. No. 85–23, revised 1996) and was approved by Animal Care and Use Committee of Nanchang University.

### Quantitative biofilm formation assay

Biofilm formation on 96-well polystyrene microtiter plates was measured by the crystal violet staining of cells cultured in LB broth as described previously [16]. Briefly, 150 μL of bacterial culture (1.5 × 10^7^ CFU/mL) was added to wells in polystyrene microtitre 96-well plates and incubated at 37 °C for 18 h. Wells were dried and measured using crystal violet (Sigma, USA) staining for 20 min. The bound dye was eluted with 95% ethanol and quantified by optical density (OD) measurement (OD_590_). Biofilm assays were performed in triplicate and repeated three times.

### RNA extraction and Illumina-based RNA sequencing

For RNA-sequencing, total RNA was extracted from WT and the TfpNDM-hvKP strains grown to log phase in LB broth with shaking at 37 °C using TRIzol reagent (Invitrogen, Paisley, UK) and purified using the RNase-free DNaseI (TaKaRa, Dalian, China) following the manufacturer’s protocol. The concentration of purified RNA was quantified by the Qubit system (Thermo Fisher Scientific). RNA quality and quantity was evaluated on a 2100 Bio-analyzer (Agilent) using the AgilentRNA 6000 Pico Kit (Agilent Technologies, USA). The ribosomal RNA was removed from total RNA using the Ribo-Zero Magnetic Kit (Bacteria) (Epicentre Biotechnologies, Madison, Wisconsin, USA). cDNA libraries were produced by the Illumina TruSeq Stranded messenger RNA Sample Preparation Kit, and sequenced on the HiSeq 2000 system as 2 × 100-bp paired-end reads. Illumina GA Pipeline (version 1.6) was used to perform the original image process to sequences, base-calling and quality value calculation.

### RNA-Seq reads mapping

To obtain the clean reads, the raw reads were filtered to low quality sequences, reads containing adapter and reads containing ploy-N. Then, clean reads were mapped to the *K. pneumoniae* BD2411 reference gene sequences using the SOAPaligner/SOAP2 (Short Oligonucleotide Alignment Program) [17]. Only mismatches with one or two bases were allowed in the alignment. The number of reads in genes was counted by using the software HTSeq-count (http://htseq.readthedocs.io/en/release_0.9.1/). Finally, the sequence results were evaluated in terms of read quality, alignment, saturation and the distribution of reads on reference genes [18].

### Identification of differentially expressed genes (DEGs)

For quantification, the gene expression levels were measured according to the number of uniquely mapped reads per kilobase of exon region per million mapped reads (RPKM) method [19]. The DESeq package was used to identify DEGs [20]. DEGs were identified using a false discovery rate (FDR) was ≤0.001 and log_2_ of the fold change (|log_2_FC|) was ≥1 as the threshold [21].

### DEG analysis and functional annotation

The functional categories of the DEGs selected above were assigned according to the KEGG (Kyoto Encyclopedia of Genes and Genomes) pathway database [22] and GO (Gene Ontology) [23] for GO and pathway annotation. Subsequently, WEGO, which is a statistical tool, was used to classify the GO annotation results for all the DEGs [24]. GO enrichment analysis of functionally significant terms in the GO database was performed using the hyper-geometric testing to determine significantly enriched GO terms among the DEGs compared to the genome background [25].

### Reverse transcriptase-quantitative PCR (RT-qPCR)

The p24835-NDM5-carrying and p24835-NDM5-free strains were grown in LB medium at 37 °C, and cells were harvested at the exponential phase of growth. A previously described method was used for assaying the gene expression [26]. Total RNA was isolated from cells using the RNeasy minikit (Qiagen), and contaminating DNA was removed by DNase I treatment using a Turbo DNA-free kit following the “Routine” protocol. RT-qPCR was performed as previously described. [27] The experiments were performed in triplicate and repeated 3 times. The relative transcript expression was determined by calculating differences in the comparative cycle threshold (Ct) method [28].

### Statistical analysis

All statistical analyses were carried out using SPSS 21.0. Continuous variables were reported as medians (25th–75th percentiles) and were analysed using the Mann-Whitney U test because data were not normally distributed. Categorical variables were expressed as frequencies (percentages) and were analysed using the chi-square test, and multiple comparisons were adjusted using the Bonferroni correction (*P* < 0.01). LD_50_ values were calculated using GraphPad Prism version 6. Survival data were analyzed with a log-rank (Mantel-Cox) test. *P*-values of < 0.05 were considered statistically significant.

## Results

### Antimicrobial susceptibility profiles

The p24835-NDM5-containing strain (TfpNDM-hvKP) was generated from the wildtype (WT) hvKP strain (BD2411). The identities of the selected derivative originated from BD2411 were compared by pulsed-field gel electrophoresis (PFGE). They had an identical PFGE pattern. The MICs of a panel of antimicrobials are presented in Additional file 3: Table S1. Results pertaining to susceptibility testing showed that the WT strain BD2411 was susceptible to most antimicrobials including carbapenems. While the transformant TfpNDM-hvKP exhibited resistance to carbapenems and harbored *bla*_NDM-5_ gene.

### Growth kinetics and fitness cost associated with p24835-NDM5 carriage

**Fig. 1:**
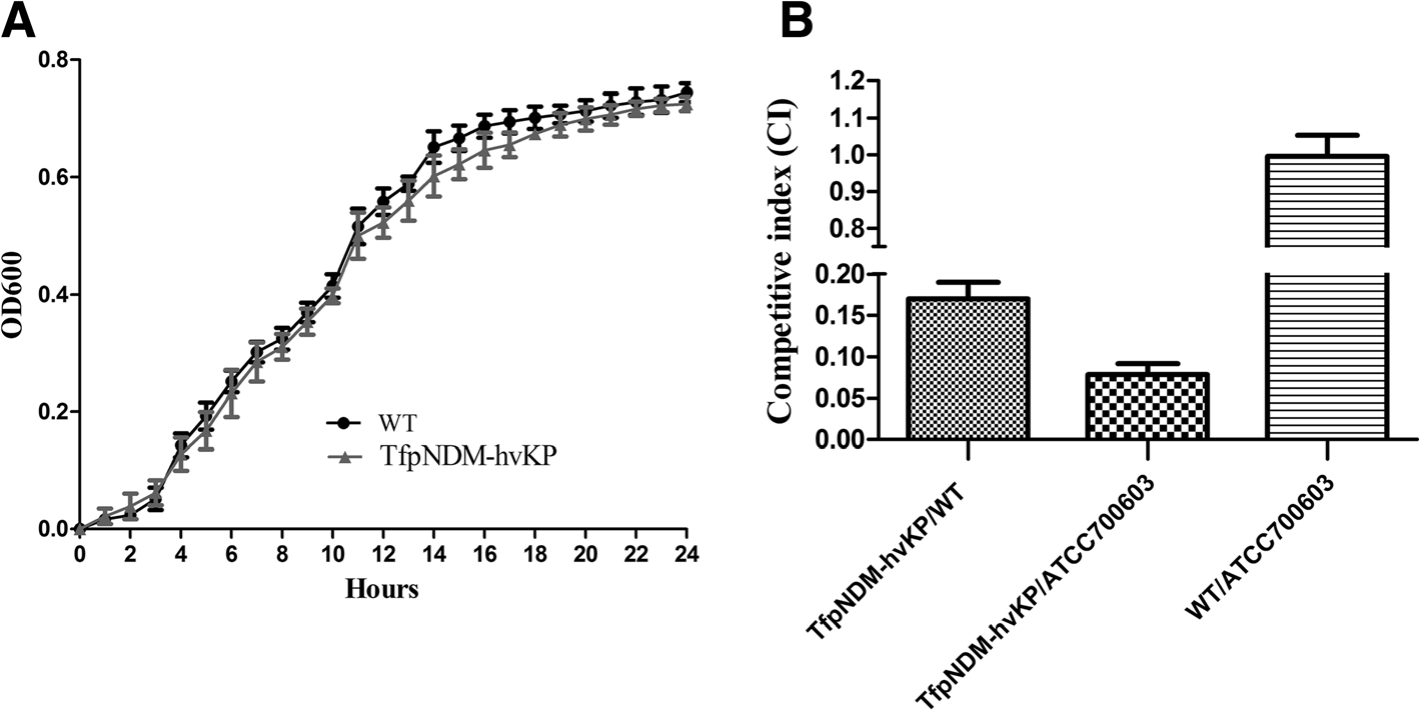
**a** Growth kinetics of the WT and its TfpNDM-hvKP strains. Values represent the mean ± standard variations obtained from three independent experiments. **b** Competitive growth kinetics. Dynamics of replicate competition experiments for *K.pneumoniae* BD2411 and its transformant containing the plasmid p24835-NDM5. The ratio of resistant versus susceptible CFU of a representative series is shown. Error bars denote the standard deviation of mean CFU counts

To investigate whether p24835-NDM5-containing strain exhibit a fitness defect compared to its plasmid-free isogenic ancestor, growth curves and competition experiments in vitro were performed. The growth rates between the transformant TfpNDM-hvKP and its plasmid-free isogenic ancestor were not significantly different (*P* = 0.94), revealing that hvKP is a suitable host to study growth rate, since the presence of p24835-NDM5 had small effect (Fig. 1a).

The CI values indicated that the p24835-NDM5-containing strain exhibited significant fitness defect when co-cultured with its plasmid-free isogenic ancestor (CI value, 0.19 ± 0.06) and the control strain ATCC700603 (CI value, 0.07 ± 0.02) (Fig. 1b). No plasmid loss was observed during the time course of the 24 h competition experiments. Furthermore, we propagated the TfpNDM-hvKP strain in monocultures for 100 generations in the absence of antibiotics, indicating that p24835-NDM5 was rapidly lost from the hvKP host strain.

### Decreased HMV phenotype and CPS production in TfpNDM-hvKP

**Fig. 2:**
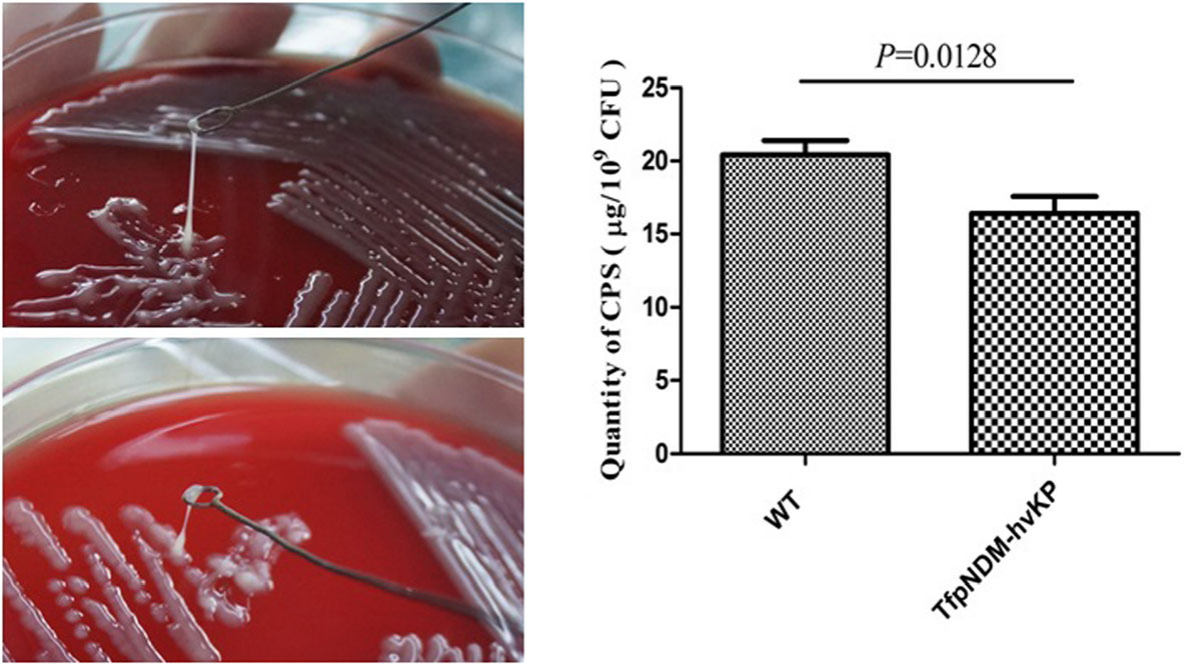
The mucoviscosity and capsular polysaccharide (CPS) production. **a** The results of mucoviscosity. The string test was used to assess the hypermucoviscosity of *K. pneumoniae* strains. A string of 5 mm or longer is defined as positive. **b** CPS production. CPS biosynthesis in the *K. pneumoniae* strains was determined by phenol-sulphuric acid assays. *P* < 0.05 versus its plasmid-free isogenic ancestor. Error bars indicate the standard deviations for three triplicate samples

Serotype K1 was identified in all strains by PCR analysis, and the HMV phenotype was confirmed in the WT strain by a positive string test (Fig. 2). In contrast, while the transformant TfpNDM-hvKP also exhibited hypermucoviscosity with a positive string test, the length of the string was reduced in comparison to its plasmid-free isogenic ancestor (Fig. 2). Consistent with the result, CPS production decreased in the transformant TfpNDM-hvKP comparing to its plasmid-free isogenic ancestor (Fig. 2).

### Reduced serum resistance and biofilm formation

**Fig. 3:**
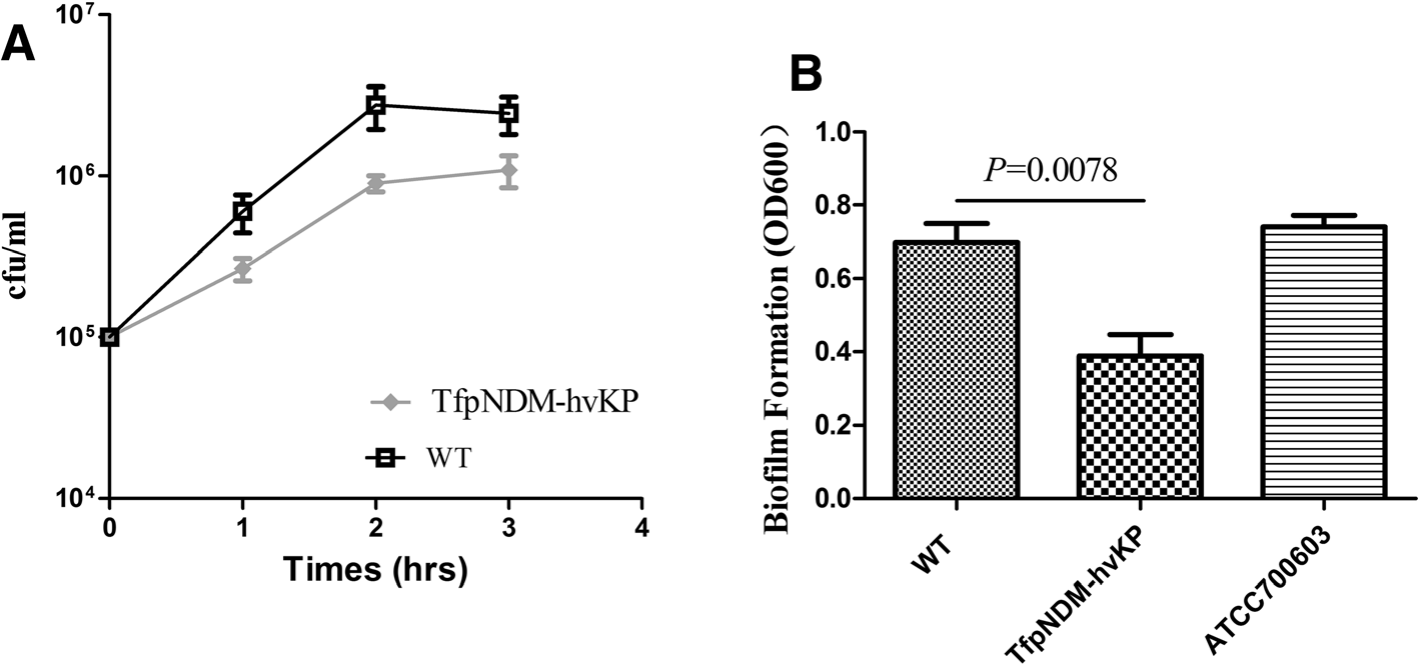
**a** Serum killing assay of the WT and its TfpNDM-hvKP strains. Data are mean ± SEM for *n* = 3. To normalize data, log_10_-transformed values were utilized, the area under each curve was calculated and compared using two-tailed unpaired t tests. (*P* > 0.05). SEM, standard error of the mean. **b** Analysis of biofilm formation by WT and its TfpNDM-hvKP strains. Overnight cultures of *K. pneumoniae* strains were grown in fresh Luria-Bertani (LB) broth at a ratio of 1:100 in polystyrene plates at 37 °C for 5 h. The bacteria were stained with crystal violet, washed to remove unbound cells, eluted with 95% ethanol, and biofilm masses were detected by measuring the absorbance at 600 nm. *P* < 0.05 versus the TfpNDM-hvKP strain. Error bars indicate the standard deviations for three triplicate samples

Survival rates of the p24835-NDM5-containing strain and its plasmid-free isogenic ancestor were evaluated by adding the normal human serum (Fig. 3a). The p24835-NDM5-containing derivative, TfpNDM-hvKP, exhibited significantly reduced survival and growth after 2 h in normal human serum compared with its p24835-NDM5-free isogenic ancestor (*P*-value < 0.05).

Figure 3b depicts the results of in vitro assays measuring biofilm formation of the transformant TfpNDM-hvKP and its plasmid-free isogenic ancestor. Compared with the control strain *K. pneumoniae* ATCC 700603, which also produces robust biofilm (OD_600_, 0.765), the wild type hypervirulent strain showed a strong biofilm-producing ability. Additionally, p24835-NDM5-containing derivative exhibited similar amounts of biofilm formation than its plasmid-free isogenic ancestor.

### High neutrophil phagocytosis and murine lethality

**Fig. 4:**
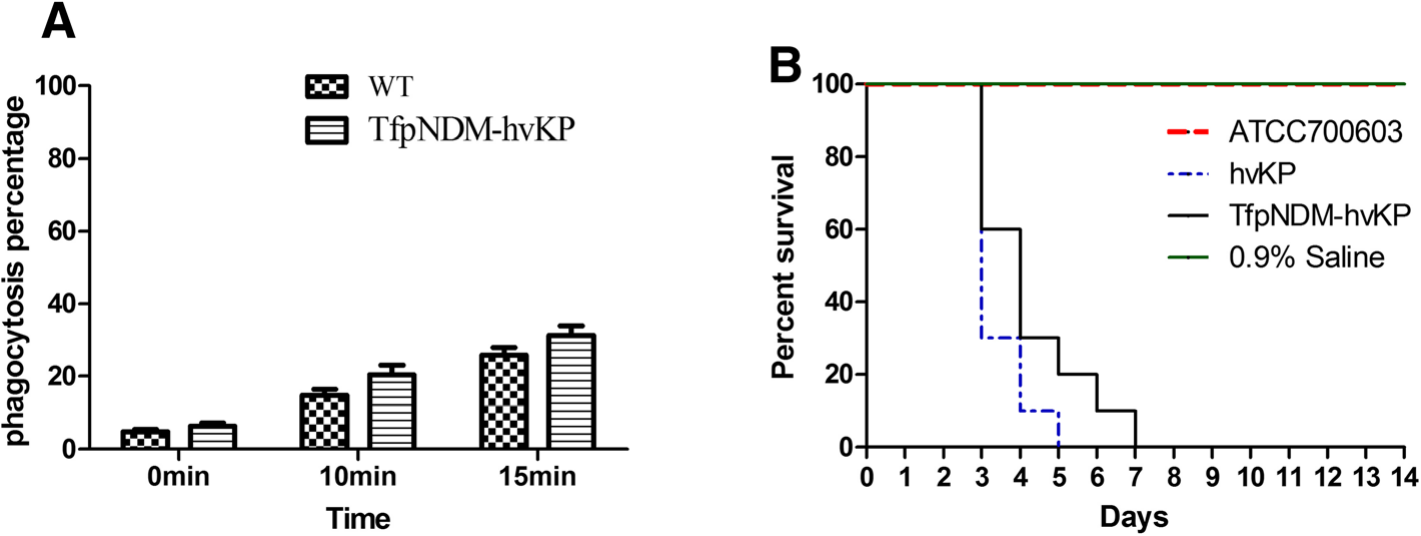
**a** Effects of the WT and its TfpNDM-hvKP strains on phagocytosis. The phagocytosis rate is calculated as the percentage of neutrophils ingesting FITC-labeled bacteria at 15 min. **b** Mouse lethality assay of the WT, its TfpNDM-hvKP, and ATCC700603 strains. The mortality of mice after intraperitoneal injection of all strains was observed over 14 days. Data points represented the percentage of mice survival in each group (*n* = 6 mice per strain)

The p24835-NDM5-containing strain developed from the p24835-NDM5-free strain showed consistent phenotypes in neutrophil phagocytosis and murine lethality (Fig. 4). The TfpNDM-hvKP strain showed a higher resistance to neutrophil phagocytosis in comparison to its plasmid-free isogenic ancestor (Fig. 4a). Murine lethality studies by intraperitoneal injection showed that LD_50_ of the TfpNDM-hvKP strain was 4.5 × 10^2^ CFU with p24835-NDM5 carriage, indicating high virulence (Fig. 4b).

### Determination and compilation of the transcriptomes of transformant TfpNDM-hvKP and its plasmid-free isogenic ancestor

**Fig. 5:**
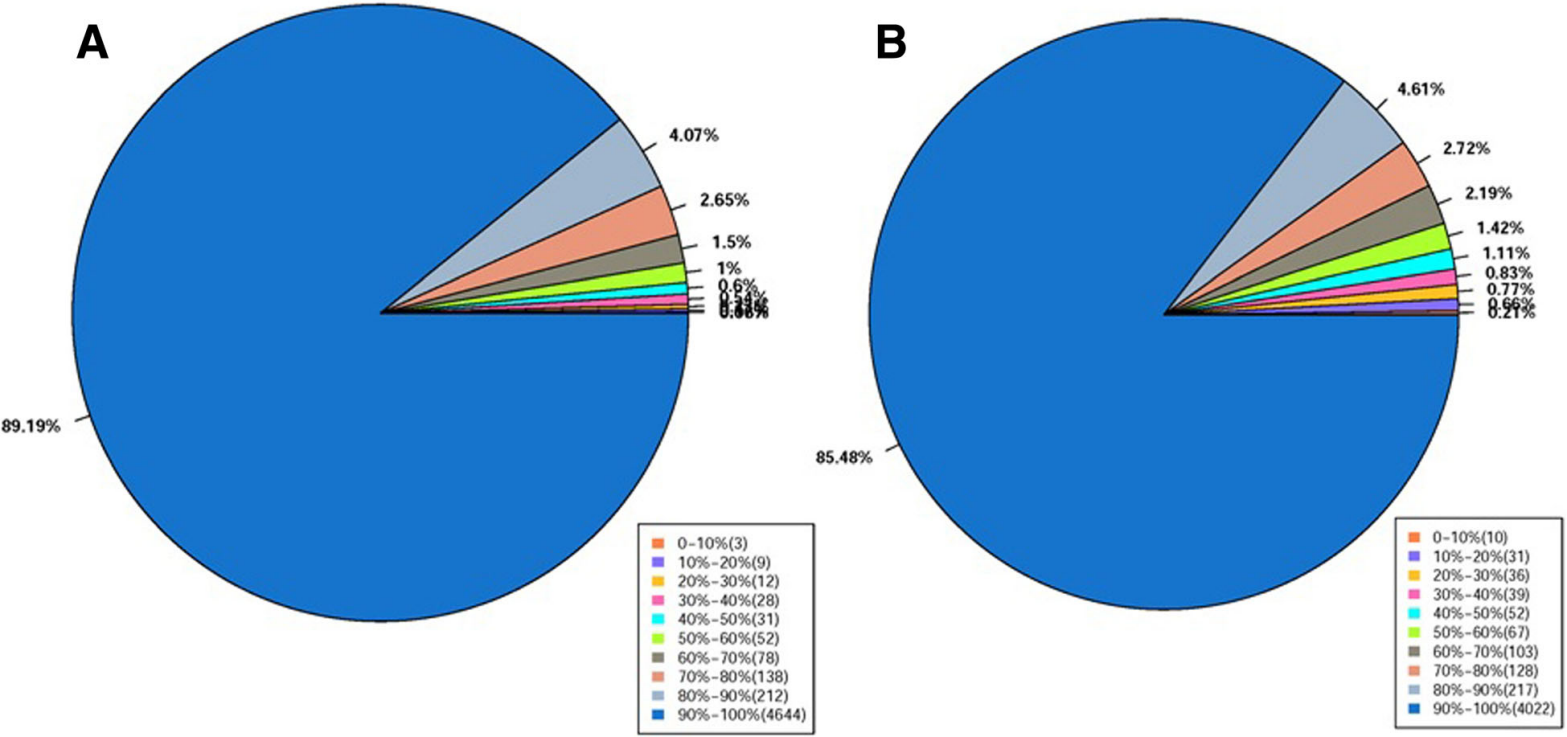
Percentage coverage in WT (**a**) and its TfpNDM-hvKP (**b**) in the assembly genome. Gene coverage is the percentage of a gene covered by reads and is equal to the ratio of base numbers in a gene covered by unique mapping reads to the total number of bases in that gene. The results are represented visually as the percentage of all genes

The total RNA from the transformant TfpNDM-hvKP and its plasmid-free isogenic ancestor was analyzed to determine the respective gene expression levels and identify differentially expressed genes. A total of 20,942,019 and 17,132,279 paired reads with lengths of 90 bases × 2 were obtained for the transformant TfpNDM-hvKP and its plasmid-free isogenic ancestor, respectively (Table 1). The quality of sequencing is indicated by the gene coverage, which is defined as the ratio of the number of bases in a gene covered by unique mapping reads to the total number of bases in that gene (Fig. 5).

**Table 1:** Summary of read numbers based on RNA-Seq data from the WT and its TfpNDM-hvKP strains

Map to gene	BD2411(WT)		BD2411 TfpNDM-hvKP	
	read number	Precentage	read number	Precentage
Total reads	20,942,019	100.00%	17,132,279	100.00%
Total base pairs	6,282,605,700	100.00%	5,139,683,700	100.00%
Total mapped reads	18,500,512	88.34%	14,414,530	84.14%
Perfect match	17,488,892	83.51%	13,172,594	76.89%
≦5 bp mismatch	1,011,620	4.83%	1,241,936	7.25%
Unique match	16,147,639	77.10%	12,222,918	71.34%
Multi-position match	352,873	1.68%	191,612	1.12%
Total unmapped reads	2,441,507	16.66%	2,717,749	15.86%

Mapped reads are the sum of perfect match reads and ≤ 5 bp mismatch reads. The percentage represents the percents of reads in each library

The transcriptomic results, obtained using RNA sequencing, were validated through the RT-qPCR analysis of a subset of differentially expressed genes as shown in Fig. 5 and the Additional file 5: Table S3. Nineteen selected genes, 7 up-regulated and 12 down-regulated genes, were well validated by RT-qPCR (Fig. 9). A good correlation was observed between the RT-qPCR data and the results obtained from the transcriptome analysis of TfpNDM-hvKP (R^2^ = 0.9153).

### The impacts of p24835-NDM5 carriage on the transcriptome of hypervirulent *K. pneumoniae*

We have carried out a comparison of WT and TfpNDM-hvKP cells transcriptome data to identify differentially expressed transcripts. Based on FDR value, a total of 683 DEGs with a > 2-fold change were identified by the comparative analysis between the WT and its TfpNDM-hvKP strains, including 107 differentially up-regulated and 576 differentially down-regulated genes (Additional file 2; Fig. 10).

**Fig. 6:**
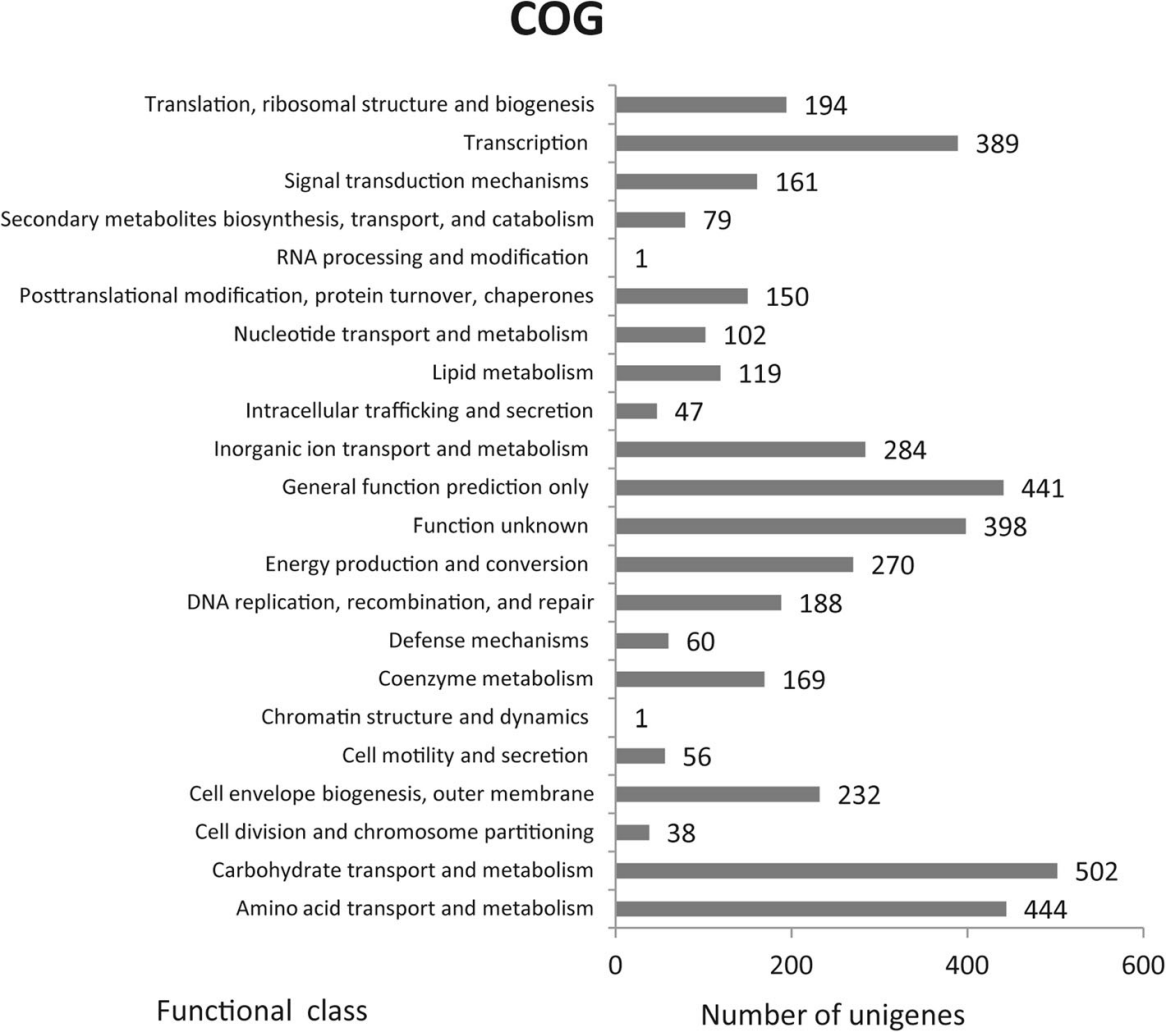
Histogram presentation of GO classification. The results were categorized into: Biological process, Molecular function, and Cellular component. The y-axis indicates the number of genes in each category

**Fig. 7:**
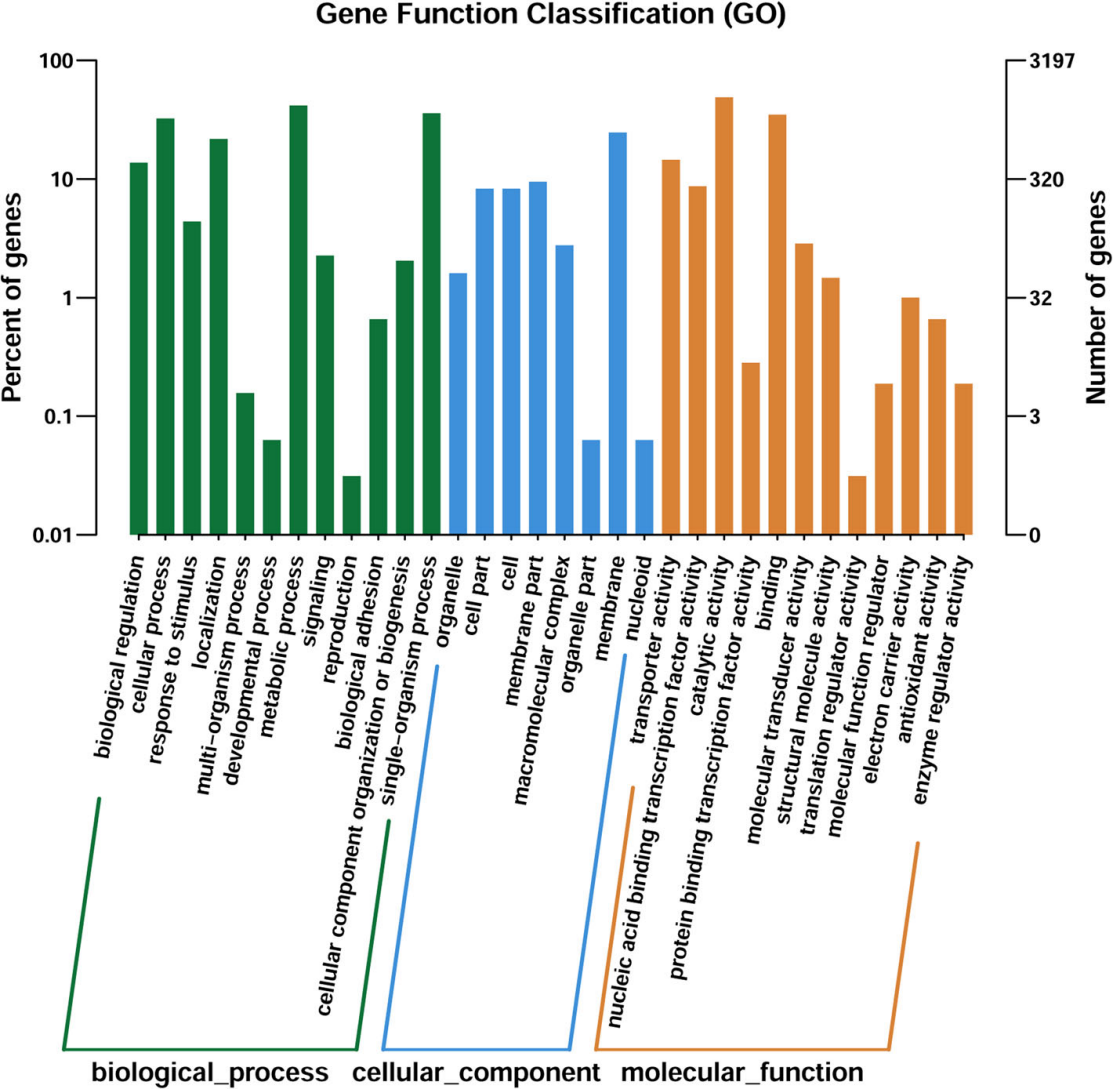
COG functional classification of the TfpNDM-hvKP transcriptome. In all, 4325 unigenes were assigned to 22 classifications. The number of unigenes is indicated to the right of each column. Any one unigene may be categorized into different classes

Based on COG annotations, the functional categories “carbohydrate transport and metabolism category”, “amino acid transport and metabolism” and “general function prediction” were significantly enriched in the unique genes in TfpNDM-hvKP (Fig. 6). However, 32 gene functional categories were enriched based on GO annotations, including “metabolic process”, “membrane”, and “catalytic activity” (Fig. 7).

**Fig. 8:**
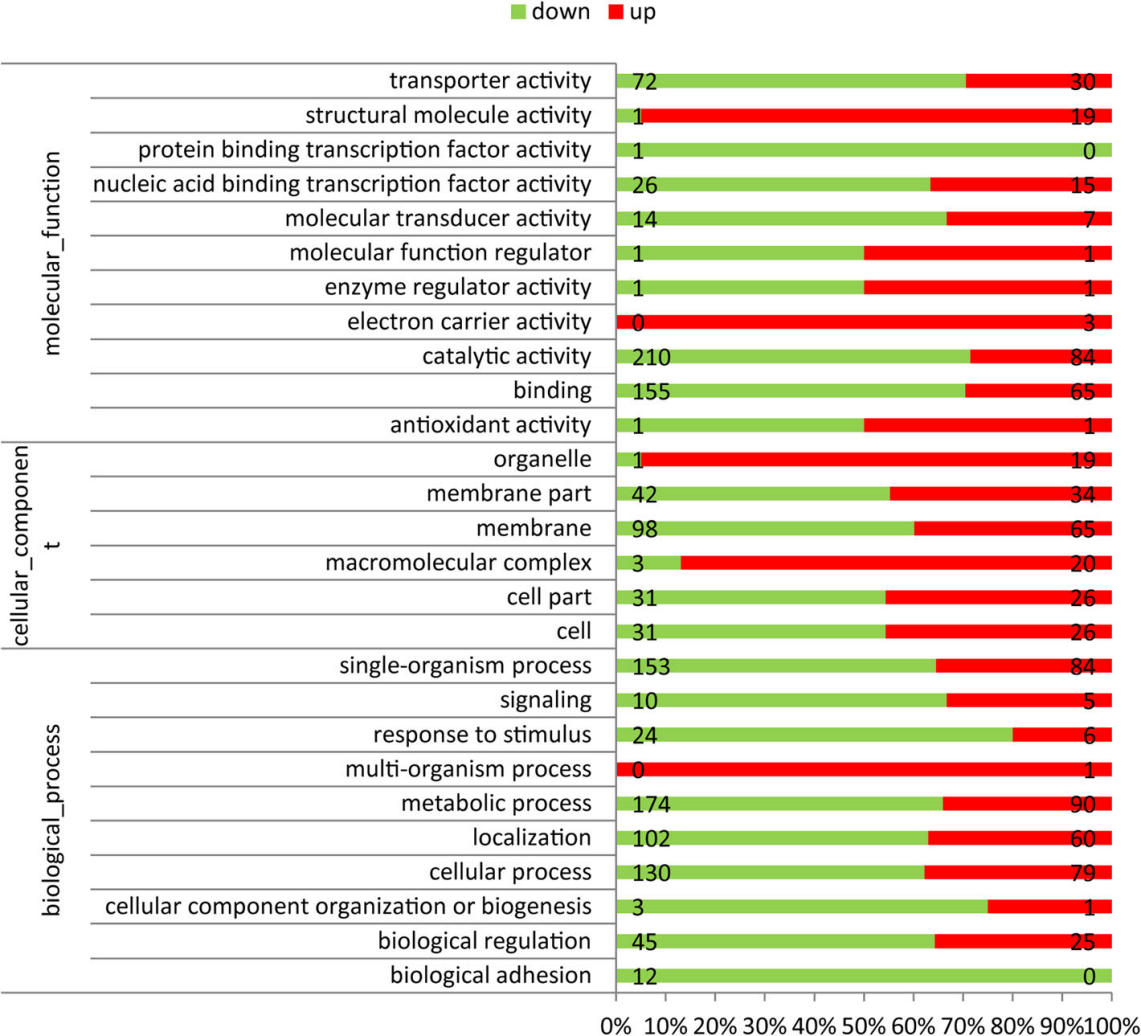
GO functional enrichment analysis of DEGs between WT and its TfpNDM-hvKP based on RNA-Seq data. Percentage of genes down-regulated in TfpNDM-hvKP compared with those in WT is indicated in green, and the percentage of up-regulated genes is indicated in red. Total number of genes differentially regulated between WT and TfpNDM-hvKP in each class is indicated on the bars

Next, a total of 683 DEGs were assigned to 95 KEGG pathways. KEGG pathway analysis showed that 45 pathways were obviously changed (*P*-value < 0.05) in the TfpNDM-hvKP strain compared with the WT strain. Notably, DEGs significantly enriched carbohydrate metabolism, cell motility, translation and xenobiotics biodegradation and metabolism (Table 2; Fig. 8).

**Table 2:** Significantly enriched KEGG pathways of DEGs incurred by carriage of p24835-NDM5 in hypervirulent *K. pneumoniae*

Pathway name	Carbohydrate metabolism	Cell motility	Translation	Xenobiotics biodegradation and metabolism
Pathway ID	ko00010	ko02030	ko03010	ko00362
DEGs with pathway annotation	86 (30.18%)	4 (1.40%)	24 (8.42%)	21 (7.37%)
All genes with pathway annotation	390 (22.49%)	8 (0.46%)	90 (5.19%)	74 (4.27%)
P value	0.000352345	0.004229524	0.00369717	0.002543
Q value	0.007399245	0.022205003	0.022205003	0.022205

In all, 285 DEGs were used for KEGG pathway annotation. KEGG pathway enrichment analysis was performed using the GOseq package. Pathways showing enrichment (Q value< 0.05) are presented

**Fig. 9:**
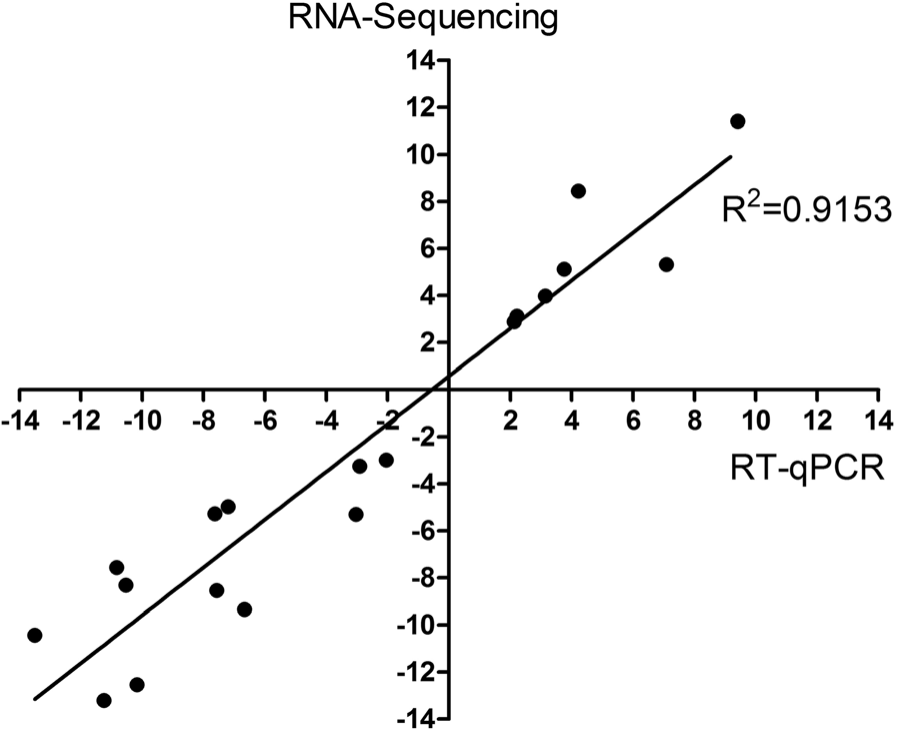
Correlations of the differential expression ratios between microarray and qRT-PCR

**Fig. 10:**
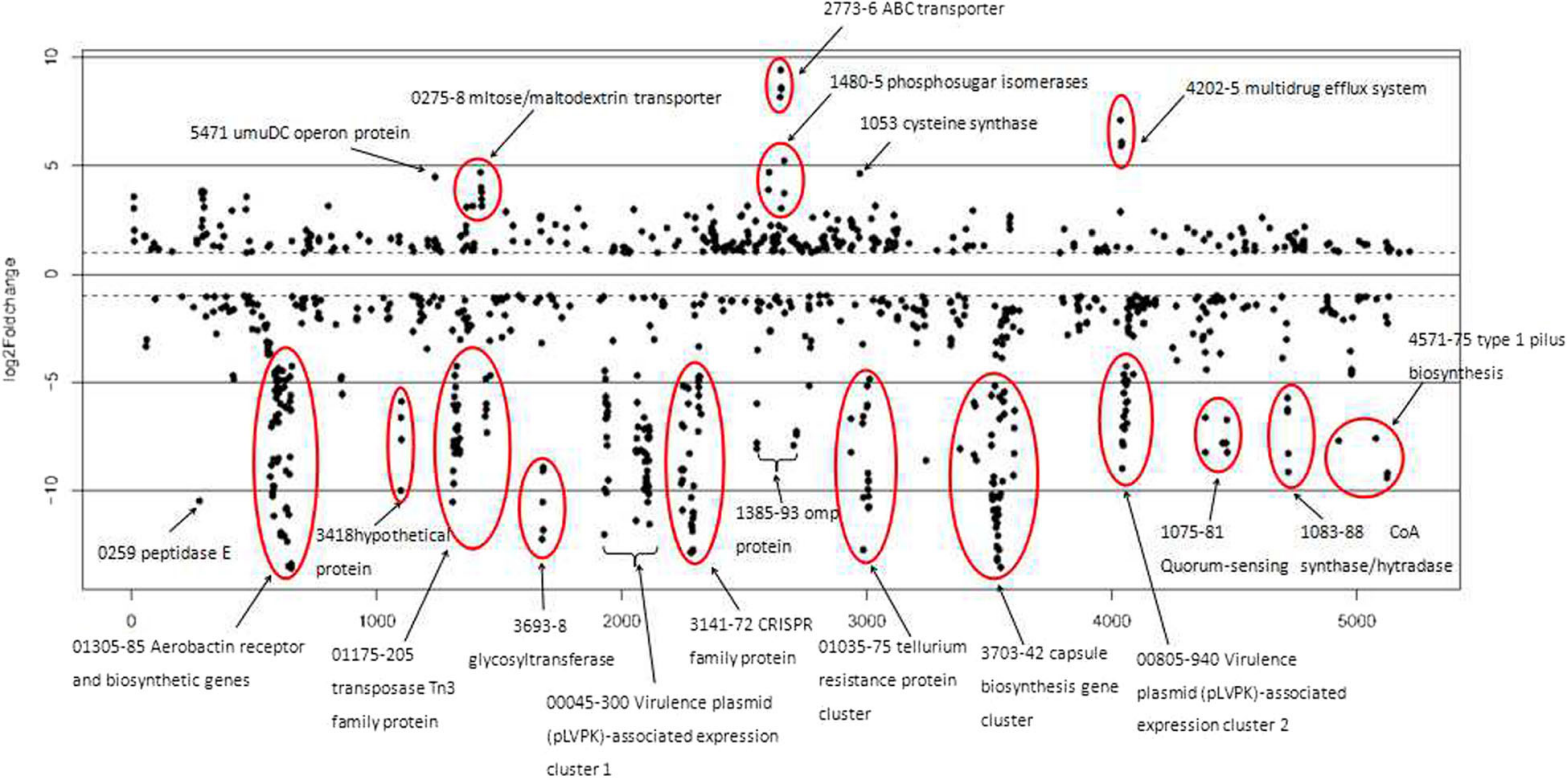
Plot of differential gene expression in WT strain compared with its TfpNDM-hvKP strain with respect to gene locus tag number. The log2 fold change in expression for each gene meeting the study threshold (log2 fold change > 1, false discovery rate < 0.001) was plotted against the gene locus tag number. Genes of interest are highlighted

### Up-regulation of multidrug efflux system and carbohydrate metabolism

Figure 5 summarizes the transcriptional responses of BD2411 associated with the p24835-NDM5 acquisition. The majority of the genes showing expression highly increased were mainly involved in carbohydrate metabolism and could cause the utilization of alternative carbon sources (Fig. 6). Strikingly, our results showed a strong correlation between the ABC- or phosphotransferase system (PTS)-dependent transporters and the enzymes that further degrade the transported carbohydrates into utilizable monosaccharides. Additionally, the genes encoding the RND family efflux transporter, sulfate transporter and putative signal peptides were overexpressed in the TfpNDM-hvKP strain, indicating that those genes might participate in pathways overlapping multidrug resistance.

### Changes in capsule biosynthesis genes expression

In the capsule locus (KP1_3714–26), the genes KP1_3714 (wzc/ptk), KP1_3718 (wzb/tyrosine kinase), KP1_3720 (wza/capsule polysaccharide export protein), and KP1_3726 (orf/glycosyltransferases) all showed decreased transcription in hvKP with p24835-NDM5 carriage. Notably, it has previously been reported that a decrease in capsule synthesis reduced expression of several genes, including wzc/ptk, which is closely related to virulence in hypervirulent *Klebsiella pneumoniae* [29]. Decreased capsule production may confer susceptible to the complement-dependent bactericidal activity, antimicrobial agents, and phagocytosis.

### Down-regulation of plasmid-borne virulence-associated genes

Figure 4 and the Additional file 4: Table S2 exhibited the transcriptional changes in the virulence-associated genes expression corresponding to their linear location on the pLVPK plasmid. Intriguingly, the plasmid pLVPK could be divided into two halves based on the transcriptional changes of the virulence-associated genes. The first half of the plasmid (with regard to tellurium resistance protein) mainly represents genes that are downregulated in response to carriage of p24835-NDM5 that can mostly be assigned to the maintenance of the heavy-metal ion homeostasis. Also, the *terZABCDE* operon, which was shown previously to be part of a pathogenicity island also containing integrase, phage, and urease genes, was down-regulated. In the second half of pLVPK, several genes involved in iron import were down regulated. Furthermore, the most adversely affected genes were *yadA* (− 11.25), coding for a plasmid-encoded adhesin, and *iutA* (− 10.17), coding for the ferric aerobactin receptor. In TfpNDM-hvKP, two siderophore-mediated iron-acquisition systems, *iucABCD, iutA* and *iroBCDN*, were down-regulated in response to p24835-NDM5 carriage.

## Discussion

*Klebsiella pneumoniae* has a notoriety for causing a wide range of infectious conditions, with numerous carbapenem-resistant strains [30]. The convergence of hypervirulence and carbapenem resistance in *K. pneumoniae* has the potential risk to cause serious global public health concern [31]. Previous studies suggested that carbapenemase-encoding plasmid not only plays a role in carbapenem resistance but also contributes to bacterial virulence [32, 33]. We wondered if the presence of the carbapenemase-encoding plasmid alters the physiology of hvKP by observing dynamic transcriptome changes.

It is commonly believed that, in the absence of antibiotic selection pressure, resistance usually imposes on the fitness cost of bacteria [34]. An obvious reduction in fitness was observed by carbapenemase plasmids and some mobile genetic elements [35]. In our study, carriage of p24835-NDM5 did not impose a burden on the growth of its hvKP host. In contrast, its carriage exerted a fitness cost. The p24835-NDM5 plasmid appears to be worse adapted to hvKP strain. A higher fitness cost was observed in the TfpNDM-hvKP compared with its plasmid-free isogenic ancestor, which might, at least in part, be explained why carbapenemase was reported less frequently in clinical isolates of hvKP [36].

The result demonstrated that the HMV phenotype was decreased in the TfpNDM-hvKP strain, and the decrease was accompanied by a markedly reduced expression of CPS. These results are consistent with the idea that the expression levels of the capsular polysaccharide synthesis related genes (eg. *magA*, *rmpA* genes) were reduced in the transformant TfpNDM-hvKP in comparison to its plasmid-free isogenic ancestor. In addition to reduced HMV and CPS production, the TfpNDM-hvKP strain exhibited increased serum sensitivity. The bactericidal effect of normal human serum is considered as a host defense system and is in part mediated by complement proteins [37]. Thus, the p24835-NDM5 carriage resulted in both carbapenem resistance and impaired many virulence-related features in the TfpNDM-hvKP strain. However, the TfpNDM-hvKP strain continued to retain its high neutrophil-mediated phagocytosis and murine lethality.

To identify the genes that were most affected by the plasmid p24835-NDM5, the variant genes in the transformant TfpNDM-hvKP and its plasmid-free isogenic ancestor were annotated using the COG, KEGG and GO databases. These annotations revealed that some functional categories were enriched in TfpNDM-hvKP compared to its plasmid-free isogenic ancestor; these categories were related to bacterial metabolism such as “carbohydrate transport and metabolism category”, “general function prediction” and “amino acid transport and metabolism”. Therefore, we propose that the presence of the plasmid p24835-NDM5 changes the physiology of hvKP by altering the transcription profiles of genes.

Previous studies suggest that plasmid containing cells enhance glucose uptake in order to produce the additional ATP needed to replicate the plasmid DNA, and to express some genes that may be located on the plasmid [38]. In our study, all strains were grown in rich Muller Hinton broth as expected, which is nutritionally rich, but not in glucose-rich conditions. The TfpNDM-hvKP strain was generally associated with up-regulation of genes related to carbohydrate metabolism and the maltose bypass. It appears that p24835-NDM5-containing cell changes its metabolism by increasing the expression of bacterial carbohydrate transporters and the homologous enzymes to make these sugars easily accessible to the pentose-phosphate pathway [39].

In this study, many virulence-associated genes located on plasmid pLVPK in hvKP strain were differentially expressed upon plasmid p24835-NDM5 acquisition. It remains to be seen whether carbapenemase-encoding plasmid might encode fundamental regulators that could alter the pathogenic process, involving invasion or secretion. The activation or deactivation of virulence genes could be associated with other plasmid-related functions. The scenario where a plasmid that induces resistance to multiple antibiotics and alters the expression of these important virulence genes is truly alarming [40, 41]. Molecular techniques such as gene knockout or super-expression should be effectively applied to identify the relationship between p24835-NDM5 carriage and each DEG.

## Conclusions

The current study defined the global transcriptional response of hypervirulent *K. pneumoniae* to plasmid p24835-NDM5. In this study, we provide an exhaustive view of the transcriptional behavior of hvKP of acquisition of *bla*_NDM_-bearing plasmid. By structuring data in clusters, we achieved a clear illustration that carbapenemase-encoding plasmid acquisition could broadly alter the metabolic pathways and physiological effects of the hvKP strain. The down-regulation of virulence-associated genes located on plasmid pLVPK was the predominant feature of this transcriptional response. It will be particularly interesting to investigate how the relationship between transcriptional changes on the plasmid pLVPK and carbapenemase-encoding plasmid acquisition at a molecular level.

Overall, the results indicated that the carbapenemase-encoding plasmid carriage not only established carbapenem resistance but also altered global gene expression in hvKP. This transcriptional study provides additional clues to understand the key molecular mechanisms involved in the acquisition of *bla*_NDM-5_-bearing plasmid of hvKP, which represents an important challenge to control dissemination of *bla*_NDM-5_ in hvKP. Moreover, the potential plasmid related transcripts identified in this study provide a good start for further investigation into the *c* adaptation in hvKP.

## Additional files

Additional file 1: **Figure S1.** Gel image of S1-PFGE result of the BD2411 and TfpNDM-hvKP isolates. (A) Isolates were digested using S1 nuclease and subjected to pulsed-field gel electrophoresis. The gel was subjected to Gel Red staining and analyzed in a CHEF-Mapper XA PFGE system. H, size marker strain *Salmonella enterica* ser. Braenderup H9812 digested with XbaI;(B) The corresponding Southern blot, hybridized with a DNA probe to the *bla*_NDM_ sequence. (PDF 143 kb)

Additional file 2: DEGs data. (XLSX 70 kb)

Additional file 3: **Table S1.** Susceptibility of *K. pnuemoniae* BD2411 (WT) and its transformant (TfpNDM-hvKP) (DOC 24 kb)

Additional file 4: **Table S2.** Comparison of differentially expressed genes in the pLVPK plasmid between the BD2411 and TfpNDM-hvKP isolates (DOC 374 kb)

Additional file 5: **Table S3.** the information and results of the 19 selected genes detected by RNA sequencing and RT-qPCR (DOC 38 kb)
