# Supplementary material for: Phenotypical profile and global transcriptomic profile of Hypervirulent Klebsiella pneumoniae due to carbapenemase-encoding plasmid acquisition
Source: BMC Genomics. 2019 Jun 11;20:480. doi: 10.1186/s12864-019-5705-2 (PMC6558890; doi:10.1186/s12864-019-5705-2)
Supplement: Supplementary file 1 — Figure S1. Gel image of S1-PFGE result of the BD2411 and TfpNDM-hvKP isolates. (A) Isolates were digested using S1 nuclease and subjected to pulsed-field gel electrophoresis. The gel was subjected to Gel Red staining and analyzed in a CHEF-Mapper XA PFGE system. H, size marker strain Salmonella enterica ser. Braenderup H9812 digested with XbaI;(B) The corresponding Southern blot, hybridized with a DNA probe to the blaNDM sequence. (PDF 143 kb) [file 12864_2019_5705_MOESM1_ESM.pdf]

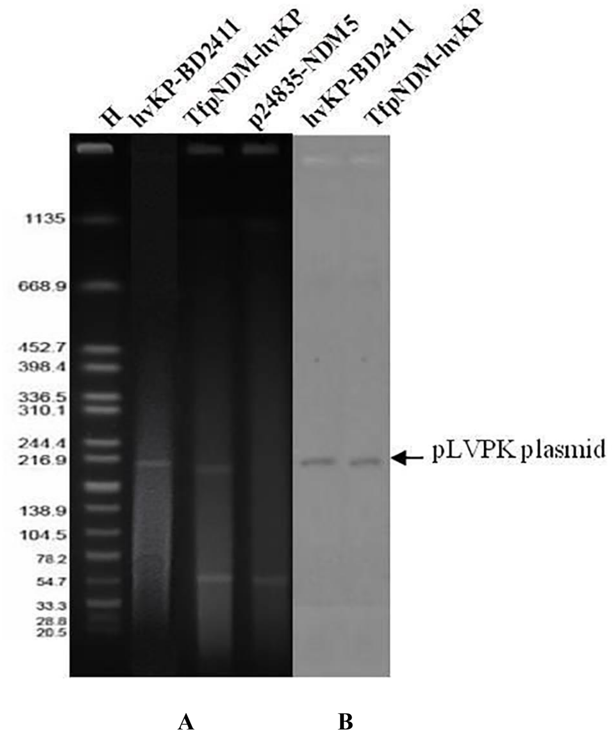

Figure S1. Gel image of S1-PFGE result of the BD2411 and TfpNDM-hvKP isolates. (A) Isolates were digested using S1 nuclease and subjected to pulsed-field gel electrophoresis. The gel was subjected to Gel Red staining and analyzed in a CHEF-Mapper XA PFGE system. H, size marker strain Salmonella enterica ser. Braenderup H9812 digested with XbaI；(B) The corresponding Southern blot, hybridized with a DNA probe to the bla_{NDM} sequence.
