# Supplementary material for: Phenotypical profile and global transcriptomic profile of Hypervirulent Klebsiella pneumoniae due to carbapenemase-encoding plasmid acquisition
Source: BMC Genomics. 2019 Jun 11;20:480. doi: 10.1186/s12864-019-5705-2 (PMC6558890; doi:10.1186/s12864-019-5705-2)
Supplement: Supplementary file 3 — Table S1. Susceptibility of K. pnuemoniae BD2411 (WT) and its transformant (TfpNDM-hvKP) (DOC 24 kb) [file 12864_2019_5705_MOESM3_ESM.doc]

Table S1 Susceptibility of *K. pnuemoniae* BD2411 (WT) and its transformant (TfpNDM-hvKP)

| Antibiotics | MIC (mg/L) | |
| --- | --- | --- |
|  | BD2411(WT) | TfpNDM-hvKP |
| Imipenem | ≦0.25 | ≧16 |
| Meropenem | ≦0.25 | 16 |
| Ceftazidime | 0.5 | 2 |
| Cefepime | 2 | 2 |
| Amikacin | ≦2 | ≦2 |
| Gentamicin | 0.5 | 1 |
| Ciprofloxacin | ≤0.06 | ≤0.06 |
| Levofloxacin | 0.25 | 0.25 |
| Ampicillin | >64 | >64 |
| Aztreonam | ≤0.06 | ≤0.06 |
| Cefotaxime | ≤0.06 | 16 |
| Piperacillin/tazobactam | 8/4 | 16/8 |
| Trimethoprim/sulfamethoxazole | 0.12/2.37 | 1/19 |
