# Supplementary material for: Phenotypical profile and global transcriptomic profile of Hypervirulent Klebsiella pneumoniae due to carbapenemase-encoding plasmid acquisition
Source: BMC Genomics. 2019 Jun 11;20:480. doi: 10.1186/s12864-019-5705-2 (PMC6558890; doi:10.1186/s12864-019-5705-2)
Supplement: Supplementary file 4 — Table S2. Comparison of differentially expressed genes in the pLVPK plasmid between the BD2411 and TfpNDM-hvKP isolates (DOC 374 kb) [file 12864_2019_5705_MOESM4_ESM.doc]

Table S2 Comparison of differentially expressed genes in the pLVPK plasmid between the BD2411 and TfpNDM-hvKP isolates

| gene | contig | strand | start | end | length | function | Expression (TfpNDM-hvKP/WT) |
| --- | --- | --- | --- | --- | --- | --- | --- |
| orf05019 | plasmid | + | 1 | 141 | 46 | conserved hypothetical protein | -3.32 |
| orf05020 | plasmid | + | 166 | 687 | 173 | RNA polymerase sigma factor FecI | 1.51 |
| orf05021 | plasmid | + | 684 | 1205 | 173 | fec operon regulator FecR | 1.42 |
| orf05022 | plasmid | - | 1610 | 1488 | 40 | conserved hypothetical protein | 2.06 |
| orf05023 | plasmid | + | 1724 | 3850 | 708 | ferric citrate outer membrane transporter | -1.74 |
| orf05024 | plasmid | + | 3925 | 4080 | 51 | hypothetical protein LV234 | -2.34 |
| orf05025 | plasmid | + | 5148 | 5591 | 147 | hypothetical protein pK2044_00180 | -1.37 |
| orf05026 | plasmid | + | 5723 | 6058 | 111 | RES domain protein | 1.68 |
| orf05027 | plasmid | + | 6140 | 6367 | 75 | IS1 repressor protein InsA | 2.47 |
| orf05028 | plasmid | + | 6544 | 6789 | 81 | hypothetical protein LV223 | 2.60 |
| orf05029 | plasmid | + | 9593 | 9796 | 67 | putative nuclease | 1.83 |
| orf05030 | plasmid | + | 10133 | 10531 | 132 | H-NS | 1.37 |
| orf05031 | plasmid | - | 12164 | 11685 | 159 | hypothetical protein LV216 | 1.82 |
| orf05032 | plasmid | - | 12346 | 12161 | 61 | YadA | -11.25 |
| orf05033 | plasmid | - | 14940 | 13165 | 591 | putative permease | -5.67 |
| orf05034 | plasmid | - | 16485 | 14959 | 508 | putative solute-binding lipoprotein in ABC transporter | 3.67 |
| orf05035 | plasmid | - | 17740 | 16496 | 414 | putative permease protein | -3.24 |
| orf05036 | plasmid | - | 18057 | 17767 | 96 | ABC transporter, ATP-binding protin | 1.34 |
| orf05037 | plasmid | - | 19226 | 18597 | 209 | ABC transporter, nucleotide binding/ATPase | 1.70 |
| orf05038 | plasmid | + | 20711 | 20857 | 48 | hypothetical protein pK2044_00290 | -8.01 |
| orf05039 | plasmid | + | 20891 | 21016 | 41 | hypothetical protein pK2044_00295 | -3.54 |
| orf05040 | plasmid | - | 21676 | 21560 | 38 | hypothetical protein EC042_pAA061 | -3.66 |
| orf05041 | plasmid | + | 22105 | 22341 | 78 | hypothetical protein LV199 | -4.29 |
| orf05042 | plasmid | + | 24047 | 25267 | 406 | CobW | -3.80 |
| orf05043 | plasmid | - | 27185 | 25536 | 549 | ferrous ion transporter protein | -4.10 |
| orf05044 | plasmid | - | 28326 | 27196 | 376 | hypothetical protein LV193 | 2.43 |
| orf05045 | plasmid | - | 28669 | 28397 | 90 | hypothetical protein pK2044_00345 | 1.52 |
| orf05046 | plasmid | - | 29707 | 28721 | 328 | hypothetical protein pK2044_00350 | -3.28 |
| orf05047 | plasmid | - | 30112 | 29858 | 84 | hypothetical protein KPN2242_25616 | -1.98 |
| orf05048 | plasmid | + | 30408 | 31607 | 399 | hypothetical protein KPN2242_25611 | -1.78 |
| orf05049 | plasmid | + | 31678 | 32100 | 140 | hypothetical protein LV190 | -2.22 |
| orf05050 | plasmid | + | 32164 | 33099 | 311 | putative GTP cyclohydrolase | 2.00 |
| orf05051 | plasmid | + | 33089 | 33649 | 186 | hypothetical protein pK2044_00375 | 1.84 |
| orf05052 | plasmid | + | 33716 | 34645 | 309 | hypothetical protein LV187 | 1.87 |
| orf05053 | plasmid | + | 35916 | 36917 | 333 | hypothetical protein pK2044_00390 | 1.87 |
| orf05054 | plasmid | + | 36877 | 37761 | 294 | hypothetical protein LV183 | 1.75 |
| orf05055 | plasmid | + | 37758 | 39104 | 448 | dihydroorotase | 1.62 |
| orf05056 | plasmid | + | 39168 | 40190 | 340 | delta-aminolevulinic acid dehydratase | 1.78 |
| orf05057 | plasmid | - | 40968 | 40756 | 70 | hypothetical protein KPN2242_25556 | -3.57 |
| orf05058 | plasmid | - | 41411 | 41016 | 131 | IS2 transposase | 4.51 |
| orf05059 | plasmid | - | 41983 | 41618 | 121 | hypothetical protein LV176 | -5.97 |
| orf05060 | plasmid | - | 43032 | 42874 | 52 | hypothetical protein KPN2242_25536 | -6.23 |
| orf05061 | plasmid | - | 43565 | 43335 | 76 | hypothetical protein LV173 | -6.57 |
| orf05062 | plasmid | - | 43782 | 43579 | 67 | hemolysin expression-modulating protein | -7.27 |
| orf05063 | plasmid | - | 44337 | 43843 | 164 | hypothetical protein LV171 | -6.45 |
| orf05064 | plasmid | - | 47117 | 44379 | 912 | TnpA transposase | -5.23 |
| orf05065 | plasmid | - | 47908 | 47351 | 185 | resolvase domain-containing protein | -7.95 |
| orf05066 | plasmid | - | 49114 | 48038 | 358 | lipoprotein signal peptidase | -6.96 |
| orf05067 | plasmid | + | 50734 | 51501 | 255 | conserved hypothetical protein | -5.17 |
| orf05068 | plasmid | - | 50745 | 49111 | 544 | Lead/cadmium-transporting ATPase (Cation efflux ATPase) | -4.92 |
| orf05069 | plasmid | - | 53732 | 52332 | 466 | copper resistant sensor kinase PcoS | -5.47 |
| orf05070 | plasmid | - | 54409 | 53729 | 226 | transcriptional regulatory protein PcoR | -3.70 |
| orf05071 | plasmid | - | 55393 | 54464 | 309 | copper resistant protein PcoD | -5.02 |
| orf05072 | plasmid | - | 55736 | 55398 | 112 | exported copper resistance protein | -6.78 |
| orf05073 | plasmid | - | 58531 | 56714 | 605 | copper resistant protein PcoA | 1.50 |
| orf05074 | plasmid | + | 58765 | 59214 | 149 | copper-binding protein | 1.62 |
| orf05075 | plasmid | - | 60471 | 60274 | 65 | hypothetical protein LV159 | 2.01 |
| orf05076 | plasmid | - | 62959 | 60512 | 815 | putative cation transporting P-type ATPase (silver resistance) | 1.42 |
| orf05077 | plasmid | - | 63526 | 63086 | 146 | hypothetical protein LV157 | 1.64 |
| orf05078 | plasmid | - | 66759 | 63613 | 1048 | SilA | -1.46 |
| orf05079 | plasmid | - | 67921 | 66770 | 383 | copper/silver efflux system membrane fusion protein CusB | -1.89 |
| orf05080 | plasmid | - | 68538 | 68176 | 120 | periplasmic copper-binding protein | 1.45 |
| orf05081 | plasmid | - | 69952 | 68567 | 461 | copper/silver efflux system outer membrane protein CusC | -7.46 |
| orf05082 | plasmid | + | 70142 | 70822 | 226 | DNA-binding transcriptional activator CusR | -7.01 |
| orf05083 | plasmid | + | 70815 | 72290 | 491 | sensor kinase CusS | -3.35 |
| orf05084 | plasmid | + | 72541 | 72972 | 143 | periplasmic Ag+-binding protein | -6.79 |
| orf05085 | plasmid | + | 73116 | 73466 | 116 | hypothetical protein LV148 | -5.19 |
| orf05086 | plasmid | - | 73841 | 73695 | 48 | hypothetical protein LV147 | -8.52 |
| orf05087 | plasmid | - | 74767 | 73853 | 304 | HNH endonuclease | -5.77 |
| orf05088 | plasmid | + | 75398 | 76372 | 324 | diguanylate cyclase | -4.15 |
| orf05089 | plasmid | + | 78168 | 78893 | 241 | phage integrase family protein | -4.11 |
| orf05090 | plasmid | - | 80024 | 79038 | 328 | putative cytoplasmic protein | -5.36 |
| orf05091 | plasmid | - | 81521 | 80553 | 322 | transposase | -4.02 |
| orf05092 | plasmid | + | 81562 | 82416 | 284 | YebB | -6.40 |
| orf05093 | plasmid | - | 84690 | 82465 | 741 | PifA | -4.02 |
| orf05094 | plasmid | - | 85594 | 84692 | 300 | PifC | -4.89 |
| orf05095 | plasmid | - | 86403 | 85879 | 174 | putative transcriptional regulator | -5.54 |
| orf05096 | plasmid | + | 86456 | 87403 | 315 | hypothetical protein LV083 | -6.90 |
| orf05097 | plasmid | + | 87739 | 88734 | 331 | transposase for IS1663 | -10.72 |
| orf05098 | plasmid | - | 89953 | 88940 | 337 | hypothetical protein LV081 | -9.21 |
| orf05099 | plasmid | + | 91435 | 93564 | 709 | hypothetical protein LV079 | -8.88 |
| orf05100 | plasmid | + | 94265 | 94381 | 38 | hypothetical protein UUU_27060 | -5.80 |
| orf05101 | plasmid | + | 94406 | 95266 | 286 | hypothetical protein LV077 | -6.03 |
| orf05102 | plasmid | + | 95421 | 95552 | 43 | putative DNA recombinase | -7.35 |
| orf05103 | plasmid | - | 99223 | 97982 | 413 | hypothetical protein LV073 | -3.70 |
| orf05104 | plasmid | - | 99883 | 99308 | 191 | TerE | -5.94 |
| orf05105 | plasmid | - | 100562 | 99984 | 192 | A Chain A, Nmr Structure And Calcium-Binding Properties Of The Tellurite Resistance Protein Terd From Klebsiella Pneumoniae | -4.76 |
| orf05106 | plasmid | - | 101641 | 100601 | 346 | TerC | -5.44 |
| orf05107 | plasmid | - | 102120 | 101665 | 151 | TerB | -6.50 |
| orf05108 | plasmid | - | 103294 | 102143 | 383 | protein TerA | -7.31 |
| orf05109 | plasmid | - | 103875 | 103291 | 194 | protein TerZ | -7.25 |
| orf05110 | plasmid | + | 104187 | 105245 | 352 | hypothetical protein PNDMMAR_p129 | -10.04 |
| orf05111 | plasmid | + | 105257 | 106399 | 380 | hypothetical protein LV064 | -10.00 |
| orf05112 | plasmid | + | 106392 | 107165 | 257 | hypothetical protein LV063 | -9.49 |
| orf05113 | plasmid | + | 107167 | 108246 | 359 | hypothetical protein LV062 | -9.77 |
| orf05114 | plasmid | + | 108246 | 109202 | 318 | hypothetical protein LV061 | -9.39 |
| orf05115 | plasmid | + | 109213 | 110436 | 407 | hypothetical protein LV060 | -9.61 |
| orf05116 | plasmid | + | 110730 | 110897 | 55 | TerW | -7.57 |
| orf05117 | plasmid | + | 112040 | 112681 | 213 | TerX | -7.72 |
| orf05118 | plasmid | + | 113413 | 114453 | 346 | hypothetical protein LV053 | -7.63 |
| orf05119 | plasmid | + | 114453 | 116186 | 577 | hypothetical protein LV054 | -7.58 |
| orf05120 | plasmid | + | 116214 | 117713 | 499 | hypothetical protein LV055 | -6.66 |
| orf05121 | plasmid | + | 118723 | 118848 | 41 | hypothetical protein KPK_A0231 | -8.24 |
| orf05122 | plasmid | - | 119522 | 119313 | 69 | hypothetical protein LV050 | -6.48 |
| orf05123 | plasmid | - | 119897 | 119685 | 70 | hypothetical protein pK2044_01015 | -9.63 |
| orf05124 | plasmid | - | 120696 | 120427 | 89 | hypothetical protein pK2044_01010 | -10.10 |
| orf05125 | plasmid | + | 120755 | 120871 | 38 | hypothetical protein pK2044_01005 | -9.14 |
| orf05126 | plasmid | - | 122604 | 120934 | 556 | hypothetical protein LV048 | -10.73 |
| orf05127 | plasmid | - | 123793 | 122585 | 402 | hypothetical protein LV049 | -12.26 |
| orf05128 | plasmid | - | 124158 | 123868 | 96 | hypothetical protein LV047 | -12.70 |
| orf05129 | plasmid | - | 125132 | 124170 | 320 | hypothetical protein LV254 | -9.57 |
| orf05130 | plasmid | - | 125692 | 125129 | 187 | hypothetical protein LV046 | -7.43 |
| orf05131 | plasmid | - | 126812 | 125703 | 369 | hypothetical protein LV045 | -5.81 |
| orf05132 | plasmid | - | 127776 | 126772 | 334 | hypothetical protein LV044 | -9.84 |
| orf05133 | plasmid | - | 128269 | 127787 | 160 | hypothetical protein pK2044_00965 | -8.92 |
| orf05134 | plasmid | + | 128636 | 130036 | 466 | hypothetical protein LV042 | -9.99 |
| orf05135 | plasmid | - | 131594 | 131202 | 130 | hypothetical protein LV041 | -7.11 |
| orf05136 | plasmid | + | 134178 | 134504 | 108 | hypothetical protein KPN2242_25901 | -7.13 |
| orf05137 | plasmid | - | 135556 | 134723 | 277 | hypothetical protein LV132 | -8.05 |
| orf05138 | plasmid | + | 135864 | 136262 | 132 | IS4 family transposase | -6.77 |
| orf05139 | plasmid | + | 136647 | 136787 | 46 | hypothetical proteinHMPREF1024_05051 | -8.67 |
| orf05140 | plasmid | - | 137106 | 136942 | 54 | putative regulatory protein | -5.38 |
| orf05141 | plasmid | + | 137207 | 137377 | 56 | hypothetical protein KP1_p216 | -4.70 |
| orf05142 | plasmid | + | 137374 | 138510 | 378 | putative recombinase | -6.34 |
| orf05143 | plasmid | - | 139182 | 139045 | 45 | hypothetical protein LV038 | -9.83 |
| orf05144 | plasmid | - | 139637 | 139365 | 90 | hypothetical protein LV037 | -7.97 |
| orf05145 | plasmid | + | 139941 | 140075 | 44 |  | -4.58 |
| orf05146 | plasmid | - | 141079 | 140120 | 319 | hypothetical protein LV035 | -5.55 |
| orf05147 | plasmid | - | 142659 | 142003 | 218 | hypothetical orf in IS2 | -9.06 |
| orf05148 | plasmid | - | 143003 | 142866 | 45 | hypothetical protein KP1_p206 | -6.40 |
| orf05149 | plasmid | + | 143246 | 144214 | 322 | transposase | 1.26 |
| orf05150 | plasmid | - | 144397 | 144224 | 57 | hypothetical protein LV030 | 3.17 |
| orf05151 | plasmid | - | 146134 | 144542 | 530 | hypothetical protein LV029 | 1.88 |
| orf05152 | plasmid | - | 146515 | 146165 | 116 | hypothetical protein LV028 | 2.09 |
| orf05153 | plasmid | - | 146700 | 146512 | 62 | Hypothetical protein B819_32473 | 1.80 |
| orf05154 | plasmid | + | 147185 | 147331 | 48 | hypothetical protein LV026 | 1.78 |
| orf05155 | plasmid | - | 149551 | 148580 | 323 | plasmid-partitioning protein | 1.62 |
| orf05156 | plasmid | - | 150717 | 149551 | 388 | SopA | 1.47 |
| orf05157 | plasmid | + | 151447 | 152457 | 336 | RepA/RepC | 1.35 |
| orf05158 | plasmid | - | 153566 | 153279 | 95 | integrase | 1.38 |
| orf05159 | plasmid | - | 153829 | 153551 | 92 | integrase | 1.60 |
| orf05160 | plasmid | - | 155417 | 155301 | 38 | hypothetical protein LV012 | -8.35 |
| orf05161 | plasmid | - | 155991 | 155524 | 155 | transposase | -9.64 |
| orf05162 | plasmid | - | 156532 | 156233 | 99 | putative transposase-related protein | -5.29 |
| orf05163 | plasmid | + | 157045 | 157428 | 127 | transposase for insertion sequence IS100 | -5.69 |
| orf05164 | plasmid | + | 157492 | 158067 | 191 | putative transposase | -3.97 |
| orf05165 | plasmid | + | 158067 | 158846 | 259 | transposase/IS protein | -5.52 |
| orf05166 | plasmid | - | 159214 | 158897 | 105 | hypothetical protein LV008 | -8.44 |
| orf05167 | plasmid | - | 159561 | 159298 | 87 | hypothetical protein LV007 | -5.04 |
| orf05168 | plasmid | - | 162661 | 161630 | 343 | putative partitioning protein ParB | -4.11 |
| orf05169 | plasmid | - | 163527 | 162661 | 288 | cobyrinic acid ac-diamide synthase | -5.08 |
| orf05170 | plasmid | + | 163747 | 163875 | 42 | hypothetical protein pK2044_00695 | -11.33 |
| orf05171 | plasmid | - | 164302 | 164051 | 83 | DnaJ-like molecular chaperone | -11.37 |
| orf05172 | plasmid | + | 164491 | 165816 | 441 | hypothetical protein LV260 | -5.11 |
| orf05173 | plasmid | + | 166029 | 166181 | 50 | conserved hypothetical protein | 1.79 |
| orf05174 | plasmid | + | 166199 | 167167 | 322 | transposase | -4.11 |
| orf05175 | plasmid | - | 167548 | 167258 | 96 | YebA | 1.78 |
| orf05176 | plasmid | - | 167898 | 167770 | 42 |  | 1.76 |
| orf05177 | plasmid | - | 168389 | 168096 | 97 | hypothetical protein LV091 | 3.54 |
| orf05178 | plasmid | - | 169538 | 168405 | 377 | hypothetical protein LV092 | 1.56 |
| orf05179 | plasmid | - | 169772 | 169644 | 42 | hypothetical protein pK2044_01250 | 3.37 |
| orf05180 | plasmid | + | 170146 | 170376 | 76 | hypothetical protein LV095 | 2.30 |
| orf05181 | plasmid | + | 170373 | 170816 | 147 | hypothetical protein LV096 | 1.01 |
| orf05182 | plasmid | - | 172797 | 172639 | 52 | hypothetical protein LV098 | -4.41 |
| orf05183 | plasmid | - | 174530 | 173610 | 306 | hypothetical protein LV101 | -7.27 |
| orf05184 | plasmid | - | 175070 | 174579 | 163 | hypothetical protein LV102a | -8.20 |
| orf05185 | plasmid | - | 175408 | 175133 | 91 | hypothetical protein LV103 | -5.72 |
| orf05186 | plasmid | - | 175920 | 175492 | 142 | hypothetical protein pK2044_01300 | -5.76 |
| orf05187 | plasmid | - | 176518 | 175958 | 186 | hypothetical protein LV105 | -4.94 |
| orf05188 | plasmid | - | 176820 | 176560 | 86 | hypothetical protein LV106 | -4.15 |
| orf05189 | plasmid | - | 179634 | 177487 | 715 | IutA | -10.17 |
| orf05190 | plasmid | - | 181047 | 179770 | 425 | IucD | -9.74 |
| orf05191 | plasmid | - | 182784 | 181051 | 577 | IucC | -7.02 |
| orf05192 | plasmid | - | 183731 | 182784 | 315 | IucB | -7.78 |
| orf05193 | plasmid | - | 185396 | 183732 | 554 | hypothetical protein pK2044_01340 | -9.15 |
| orf05194 | plasmid | + | 185589 | 186812 | 407 | ShiF | -2.33 |
| orf05195 | plasmid | + | 187510 | 187704 | 64 | hypothetical protein pK2044_01355 | -1.52 |
| orf05196 | plasmid | + | 188046 | 188381 | 111 | conserved hypothetical protein | -1.25 |
| orf05197 | plasmid | + | 188565 | 189074 | 169 | peptide deformylase | -1.68 |
| orf05198 | plasmid | - | 189941 | 189624 | 105 | lysozyme inhibitor | 1.03 |
| orf05199 | plasmid | + | 190662 | 191861 | 399 | hypothetical protein LV120 | 1.24 |
| orf05200 | plasmid | - | 194672 | 193638 | 344 | ABC transporter permease protein | 1.23 |
| orf05201 | plasmid | - | 195454 | 194642 | 270 | hypothetical protein pK2044_00025 | 1.23 |
| orf05202 | plasmid | + | 196647 | 196895 | 82 | hypothetical protein LV128 | 1.35 |
| orf05203 | plasmid | + | 197147 | 198115 | 322 | transposase | -1.24 |
| orf05204 | plasmid | + | 198306 | 198545 | 79 | hypothetical protein KP1_p013 | 2.49 |
| orf05205 | plasmid | + | 199387 | 199551 | 54 | isrso11-transposase orfb protein | -5.95 |
| orf05206 | plasmid | - | 199995 | 199729 | 88 | hypothetical protein KPN2242_26181 | -4.76 |
| orf05207 | plasmid | + | 201579 | 201737 | 52 | hypothetical protein KP1_p021 | -3.89 |
| orf05208 | plasmid | - | 205964 | 203892 | 690 | IroN | -2.19 |
| orf05209 | plasmid | - | 207746 | 206526 | 406 | IroD | -4.05 |
| orf05210 | plasmid | - | 211348 | 207860 | 1162 | IroC | -3.25 |
| orf05211 | plasmid | - | 212714 | 211647 | 355 | IroB | -2.58 |
| orf05212 | plasmid | - | 213756 | 213112 | 214 | hypothetical protein LV239 | -1.27 |
| orf05213 | plasmid | - | 214973 | 213741 | 410 | hypothetical protein LV238 | -1.40 |
| orf05214 | plasmid | - | 215552 | 215430 | 40 | hypothetical protein pK2044_00135 | 1.09 |
