# Supplementary material for: Phenotypical profile and global transcriptomic profile of Hypervirulent Klebsiella pneumoniae due to carbapenemase-encoding plasmid acquisition
Source: BMC Genomics. 2019 Jun 11;20:480. doi: 10.1186/s12864-019-5705-2 (PMC6558890; doi:10.1186/s12864-019-5705-2)
Supplement: Supplementary file 5 — Table S3. the information and results of the 19 selected genes detected by RNA sequencing and RT-qPCR (DOC 38 kb) [file 12864_2019_5705_MOESM5_ESM.doc]

Table S3 The information and results of the 19 selected genes detected by RNA sequencing and RT-qPCR

| Gene tag | Protein number | Protein description | RNA sequencing | RT-qPCR |
| --- | --- | --- | --- | --- |
| KP1_2774 | 1023 | periplasmic binding protein/LacI transcriptional regulator | 9.41 | 11.4 |
| KP1_4202 | 363 | AraC family transcriptional regulator | 7.09 | 5.32 |
| pK2044_00820 | 183 | umuDC operon protein-like protein | 4.22 | 8.45 |
| KP1_0277 | 1290 | maltoporin | 3.76 | 5.12 |
| KP1_3196 | 2154 | TonB-dependent receptor | 3.14 | 3.97 |
| KP1_1611 | 294 | cold shock protein CspE | 2.21 | 3.12 |
| KP1_4739 | 204 | glycogen synthesis protein GlgS | 2.12 | 2.89 |
| KP1_3726 | 897 | UTP--glucose-1-phosphate uridylyltransferase subunit GalF | -13.50 | -10.43 |
| pK2044_00945 | 453 | YadA | -11.25 | -13.21 |
| KP1_3714 | 1227 | rmpA | -10.83 | -7.56 |
| KP1_1357 | 924 | DNA-binding transcriptional activator AllS | -10.53 | -8.32 |
| pK2044_01320 | 2202 | IutA | -10.17 | -12.54 |
| pK2044_00100 | 2175 | outer membrane receptor FepA | -7.63 | -5.28 |
| pK2044_01075 | 459 | TerW | -7.57 | -8.54 |
| KP1_1375 | 1668 | membrane protein FdrA | -7.20 | -4.97 |
| KP1_2210 | 882 | LysR family transcriptional regulator | -3.03 | -5.31 |
| KP1_3745 | 1416 | multidrug efflux system protein MdtE | -2.91 | -3.25 |
| KP1_0790 | 621 | periplasmic protein | -2.04 | -2.99 |
| KP1_3720 | 1134 | magA | -6.67 | -9.34 |
